# Assessment of Septoplasty Effectiveness using Acoustic Rhinometry and Rhinomanometry

**Published:** 2013

**Authors:** Mohammad Hossein Dadgarnia, Mohammad Hossein Baradaranfar, Mona Mazidi, Seyed Mohammad Reza Azimi meibodi

**Affiliations:** 1 *Rhinology Research Center, Department of Otorhinolaryngology, Shahid Sadoughi University of Medical Sciences, Yazd, Iran.*

**Keywords:** Acoustic rhinometry, Nasal airway obstruction, Nasal septum, Rhinomanometry

## Abstract

**Introduction:**

Septal deviation is the chief cause of chronic nasal obstruction. In order to treat such cases, nasal septoplasty surgery is usually performed based on patient complaints and a surgeon's examination, both of which are subjective. This study aims at using the objective parameters of acoustic rhinometry and rhinomanometry to evaluate the effectiveness of septoplasty surgery.

**Materials and Methods:**

A prospective study was performed in 30 candidate patients for septoplasty surgery. Acoustic rhinometry and rhinomanometry tests were performed on all patients both before and 3 months after the operation. The symptom recovery rate was recorded according to the patient's statements and anterior rhinoscopic examinations 3 months after surgery. Data were analyzed using a t-test and chi-square tests in a SPSS package.

**Results:**

A total of 26 of 30 patients returned for a post–procedure follow-up examination after 3 months. Patients were aged from 18 to 32 years (average, 25 years). In total 69.2% (18 patients) were satisfied with the results of the procedure. In addition, rhinomanometry resulted in a decrease in general nasal resistance if patients used decongestants (P=0.03). However, the decrease was not significant before the use of decongestants (P=0.12). Furthermore, according to the results from acoustic rhinomanometry, there was an increase in the nasal cross-sectional area on both the narrow and wide sides after the operation (P<0.05), although this increase was not so notable in the narrower side after using decongestants. There was, however, no significant relationship between the results from the objective tests and the patient's symptoms or clinical examinations (P>0.05).

**Conclusion:**

The findings of this study show that although the objective tests confirm an improvement in general nasal resistance and an increase in the nasal cross-sectional area after surgery, no unambiguous relationship between the patient's symptoms and the clinical examinations is observed. Therefore, such objective tests do not prove to be sufficient diagnostic criteria for the effectiveness of septoplasty.

## Introduction

Nasal obstruction is the most common complaint among patients suffering from sinonasal diseases (1). In a study on a random sample of 300 adult patients, 33% of whom suffered from either chronic or recurrent respiratory problems, 26% had septal deformities (2). Nasal obstruction symptoms may be rooted in a number of underlying problems including nasal congestion, turbinate hypertrophy, adenoid hypertrophy, and nasal polyps for example. However, septal deviation is the most common constructional cause of such a problem (3, 4). Generally, there are four nasal airway limitations, including external nasal meatus, internal nasal meatus, nasal septum, and turbinate. The caudal margin of the nasal septum is the first air contact area, causing disturbance in the air and dividing it (5). A slight deviation in the anterior of the septum can produce obvious symptoms, whereas a severe deviation in the posterior produces milder symptoms (6). The specialized surgery intended to correct septal deviations is a well-recognized procedure called septoplasty. Septoplasty is the third most common surgical procedure carried out by ear, nose, and throat (ENT) specialists in the USA, and is most often performed in order to improve the life quality of patients (7).

Nasal obstruction symptoms are relatively subjective and cannot typically be assessed clinically. Therefore, alternative objective criteria are used to evaluate the openness of the nasal airway. Numerous methods and instruments have been introduced for this purpose; however, rhinomanometry and acoustic rhinometry are the most significant. Indeed, since 1950, rhinomanometry has been used for the objective diagnosis of nasal obstructions. In this method, nasal airflow and pressure reduction between the nasopharynx and the nasal anterior area are measured simultaneously, and nasal resistance is calculated (8). In the past 10 years, rhinomanometry has increasingly been used due to of the greater availability of microcomputers which may be connected to the measuring instruments. Using these devices, all mathematical analyses can be performed in a matter of a few seconds (9,10). Although numerous studies have been conducted into rhinomanometry, few have been sufficiently scientifically rigorous, and the clinical application of this device is still in dispute among physicians (11).

An acoustic rhinometry instrument is a device which is designed for the objective evaluation and measurement of the nasal cross-sectional area and volume, from the nasal meatus to the nasopharynx (12). Computer programs are used to analyze the sound waves reflected from the inside of the nasal meatus. The device can evaluate construction abnormalities and the physiological conditions of the nasal meatus. Thus, both of the devices mentioned above are designed to measure the degree of openness from two different views. 

The outcomes of septoplasty surgery for the treatment of septal deviations and the effects of the procedure on quality of life have not yet been unequivocally demonstrated. Although there have been some descriptive studies on objective and subjective measurements after septoplasty, the studies have mostly been retrospective (13–15).

Therefore, the present study aims at the objective evaluation of the effectiveness of nasal septoplasty as a treatment for patients diagnosed with septal deviations, in addition to providing evidence for variations in the nasal airway through objective methods such as rhinomanometry and acoustic rhinometry.

## Materials and Methods

This prospective study was carried out in 30 patients who had nasal obstruction complaints and had been referred to ENT clinics at Shahid Sadughi and Shahid Rahnemun Hospitals in Yazd from 2008 to 2010. Patients were asked about their medical history before undergoing anterior rhinoscopic examination. Based on the information collected, some candidate patients were selected for surgery. Subjective symptoms of nasal obstruction before and after surgery were recorded on a visual analogue scale, and classified as mild, moderate, or severe. Patients who suffered from allergic rhinitis, tumors, nasal polyps, complete nasal obstruction in one side, or hypothyroidism and those who used to smoke or take aspirin were excluded from the study. 

Next, patients were kept under objective observation using an acoustic rhinometry A1 and rhinomanometry NR6 instrument. In the rhinomanometry test, the instrument was first calibrated. According to the international standard committee's regulations in 2005(16), rhinomanometry was carried out in a standard, active, anterior procedure at a certain time during the day (8 am). Patients were placed in a seated position and asked to breathe with their mouths closed. The computer plotted a graph and calculated the nasal resistance on both sides as well as the total nasal resistance. Then, by changing the location of the pressure convertor pipe, these computations were also performed on the opposite side. All of the results were evaluated at a pressure of 150 Pa. 

After completion of this test, two pieces of cotton soaked in phenylephrine were placed into the patient's nose for 10 mins as a decongestant. The same test procedures were then repeated. 

As for the acoustic rhinometry test, the instrument was first calibrated using an acoustic wave. A nasal piece was placed on the nasal meatus in a parallel position to the nose and held there without causing any deformity to the nose. The patients were asked to hold their breath during the test. The computer plotted the results and calculated the nasal cross-sectional area on both the left and right sides as well as the total nasal cross-sectional area. The test was also performed for the opposite side, and was repeated after using two pieces of phenylephrine as nasal decongestants to minimize the effect of the nasal cycle.

The results of the rhinometry test were classified into low-resistance (<0.25) and high-resistance (>0.25) groups before using decongestants, and high-resistance (>0.18) and low-resistance (<0.18) groups after use of decongestants. With regard to acoustic rhinometry, these groups proved to have small cross-sectional areas (< 0.73) and large cross-sectional areas (>0.73) before using decongestants. There were also two groups of narrow cross-sectional area (<0.92) and wide cross-sectional area (>0.92) after use of decongestants (17).

Three months after the operation, 26 patients returned for follow-up examinations and were subjected to rhinomanometry and acoustic rhinometry tests. In addition to a clinical examination of the patients' noses by anterior rhinoscopy, their degree of satisfaction was recorded after surgery, as well as improvements in their nasal obstruction symptoms. Finally, the collected data were analyzed using chi-square tests and paired t-tests.

## Results

In this study, 30 patients aged from 18 to 32 years (average, 25 years) were considered candidates for septoplasty surgery. Four patients (13.3%) did not return for follow-up examinations. Among the 26 patients who did return and were evaluated after surgery, 73.1% (19 patients) were male and 26.9% (seven patients) were female. 

In total, 7.7% of the patients had mild symptoms, 23.1% had moderate symptoms, and 69.2% had severe symptoms of nasal obstruction before surgery. After surgery, 38.5% reported no symptoms, 34.4% reported mild symptoms, and 26.9% reported severe symptoms. A total of 18 out of 26 patients who entered the study were satisfied with the outcomes of the surgery, while eight were not satisfied. In the rhinoscopic clinical examination before surgery, 3.8% of patients had mild septal deviations, 46.2% had moderate deviations, and 50% had severe deviations. However, after surgery, 80.8% of patients had no or mild deviations and 19.2% had moderate or severe deviations. In order to find the relationship between objective tests and patients' symptoms, the results were classified based on the cut-off points under consideration (11). The evaluation of general nasal resistance through a rhinomanometry test did not reveal any significant statistical difference before and after the operation without use of decongestants (P=0.21). However after the use of decongestants, a significant decrease in general nasal resistance was observed following surgery compared with pre-surgical values (P=0.03) (Table 1).

**Table 1:** Rhinomanometry test results before and after surgery**.**

	Before decongestant		After decongestant	
	After operation	Before operation	After operation	Before operation
Total Nasal Resistance	0.49±0.54	0.85± 0.78	0.3 ±0.41	0.84 ±0.71
**P-value**	**0.21**		**0.03**	

In the acoustic rhinomanometry test, the cross-sectional areas of narrow and wide nasal meatus were compared before and after surgery. The results revealed that before using the decongestants, the increase in the nasal cross-sectional area on both the narrow and wide sides was statistically significant after surgery (P=0.009 and P=0.43, respectively). However, after using decongestants, it was only the wider side of the nose that showed a significant increase in its cross-sectional area (P=0.012); such an increase was not significant on the narrower side (Tables 2 and 3).

**Table 2:** Results of acoustic rhinometry on the wider side of the nose before and after surgery

	**Before decongestant**		**After decongestant**		
	Before operation	After operation	Before operation		After operation
Wider side	1.06±0.99	0.93±1.27	0.75±0.86		0.8±1.23
P-value	0.009			0.145	

Pair t _test

**Table 3:** Results of acoustic rhinometry on the narrower side of the nose before and after surgery

	**Before decongestant**		**After decongestant**		
	Before operation	After operation	Before operation		After operation
Narrower side	0.37±0.73	0.78±1.47	0.8±0.92		0.92±1.27
P-value	0.043			0.012	

In general, 69.2% of patients (18 patients) were satisfied with their symptomatic improvement after surgery, whereas 30.8% (eight patients) did not report any improvement in symptoms. A total of 11.7% of patients who underwent septoplasty surgery still complained about nasal obstruction following the operation, despite an improvement in rhinomanometry results; however, after using decongestants, this number reduced to 9%. Among the patients whose acoustic rhinometry test results had improved, the dissatisfaction rate was 4.7% on the narrower side and 5.5% on the wider side. However, the satisfaction rate of patients in relation to their recovery from symptoms did not prove to have any statistically significant relationship with the objective tests results (Table 4 and 5).

**Table 4:** Comparison between rhinomanometry test results after surgery (both before and after using decongestant) and patients' satisfaction rate

Rhinomanometry results Patients satisfaction	**Before decongestant**		**After decongestant**	
	Low resistance	High resistance	Low resistance	High resistance
Yes	22.2%	77.8%	5.5%	%94/5
No	25%	75%	0%	100%
P-value	0.87		0.49	

**Table 5:** Comparison between acoustic rhinometry test results after surgery (both before and after using decongestant) and patients' satisfaction rate

Acoustic	**Before decongestant,** **wider side**		**After decongestant, narrower side**		**Before decongestant, wider side**		**After decongestant, narrower side**	
Rhinometry results Patients satisfaction	wide cross-sectional area	Narrow cross-sectional area	wide cross-sectional area	Narrow cross-sectional area	wide cross-sectional area	Narrow cross-sectional area	wide cross-sectional area	Narrow cross-sectional area
Yes	72.2%	27.8%	66.7%	33.3%	66.7%	33.3%	50%	50%
No	75%	25%	87.5%	12.5%	75%	25%	75%	25%
P-value	0.88		0.26		0.67		0.23	

## Discussion

Nasal obstruction is one of the most common complaints among patients referring to ENT specialists, and septoplasty is typically carried out for the treatment of such a condition. An objective evaluation of the openness of the nasal airway is considered necessary in modern respiratory medicine. The best-known methods used for this objective evaluation are rhinomanometry and acoustic rhinometry. Acoustic rhinometry is more applicable to cases of secondary obstruction with constructive abnormalities. Rhinomanometry is a high priority for patients suffering from functional nasal obstruction condition such as allergic rhinitis (18). 

The results of the present study indicate that nasal resistance as measured by rhinomanometry significantly deceases after septoplasty. Moreover, a significant increase in the nasal cross-sectional area and volume was revealed through acoustic rhinometry after surgery. Such an increase in the nasal cross-sectional area was observed on the narrower side before using a decongestant and in the wider side both before and after using a decongestant. Brom et al. observed a significant decrease in nasal airway resistance before and after surgery (15). Similarly, Gordon et al. witnessed a considerable decrease in resistance after surgery (19). In Lio's study, researchers observed an improvement in resistance of the cross-sectional area on the narrower side after septoplasty, while no variations were witnessed on the wider side. Lio concluded that septoplasty leads to an improvement in the nasal performance on the afflicted side and no special affliction on the healthy side (20). Kemker investigated septoplasty effectiveness on acoustic rhinometry results only and discovered a considerable increase in the nasal cross-sectional area and volume, particularly in the more posterior portions after surgery. In addition, he found no relationship between objective tests results and clinical findings or symptoms (21).

In the study by Prilia et al. (22), a decreased cross-sectional area was observed in MCA1 (the smallest part in the nasal cross-sectional area in meatus) on the side with deviation. A significant decrease was also observed in the MCA1 on the opposite side, both before and after using a decongestant after surgery. In an evaluation of MCA2 (the second smallest nasal cross-sectional area in meatus), there was a significant increase on the deviate side before and after using a decongestant following surgery; however, the increase on the other side was not considerable. Since the cross-sectional area reaches a minimum in the nasal meatus, any correction of the nasal deviation in the anterior area would be more effective then corrections in the posterior region. As can be seen, there is an improvement in rhinomanometric and acoustic rhinometric parameters on the afflicted side in studies similar to the present study. 

In the present study, 69.2% of patients were satisfied with the surgery. The patients' dissatisfaction rate was 11%, despite improvements in the rhinomanometry results, and was 4.7–5.5% in the acoustic rhinometry test. However, there was no significant relationship between the objective test results and the patients' satisfaction or clinical symptoms. As reported in a number of previous studies, patients' satisfaction rate after surgery is 72–90%, which indicates that septoplasty is effective in reducing nasal obstruction.

Kim et al. (1) did not observe any significant relationship between changes in patients' symptoms and nasal resistance or the cross-sectional area. In their study, Tomkinson and Eccles (23) could find only a limited relationship between patients' complaints and acoustic rhinometry results; however, there was a stronger relationship between acoustic rhinometry data and the results of CT, MRI, and anterior rhinoscopy.

Gordon (19) reported that 22% of the patients who had undergone a septoplasty procedure still had complaints about nasal obstruction after the surgery, despite improvements in their rhinomanometry results. In another study, Chung Seop (24) did not observe any relationship between nasal resistance and variations in the cross-sectional area on one hand and patients' symptoms on the other. As a result, he concluded that such objective tests do not offer any diagnostic value with respect to evaluating the severity of nasal construction symptoms.

In the study by Pirila et al. (22), 65% of the patients confirmed complete or fairly good satisfaction with the outcomes of the surgery and just 10% reported little satisfaction. There was a notable relationship between the patients' satisfaction and the increase in MCA1 on the deviant side after using a decongestant, although the positive relationship was not significant between the patients' satisfaction rate and the increase in airflow, nasal volume, or MCA1. Furthermore, an opposite relationship was observed between the decrease in the cross-sectional area and the patients' satisfaction after surgery, but it was not significant. Therefore, a significant relationship existed in the evaluation of the nose on both sides, between satisfaction after surgery and increase in MCA1. However, such a relationship was not noticeable with other acoustic rhinometric parameters. Furthermore, the relationship between the clinical examinations of the nose and rhinomanometry and acoustic rhinometric parameters was not considerable. In this study, Prilia evaluated two cross-sectional areas of the nose, and MCA1 was more affected by surgery. On the other hand, a general evaluation of the nose has been carried out in this present study. Clearly, there is a possibility of errors in the measurement of MCA1 due to software errors in setting a zero point.

Lam et al. (25) found no significant relationship between the objective and subjective measurement of nasal obstruction. The effect of pre-operation factors on

patients' satisfaction after the operation has been explored in only a few studies. In Sipila's study (26), it was observed that the greater the pre-operation resistance in rhinomanometry, the higher the satisfaction after surgery. However, Dinis (27) found no relationship between surgery effectiveness and nasal resistance after surgery. 

In the study by Brom et al. (15), 17% of patients showed no decrease in nasal resistance after surgery, while 26% reported recovery from the symptoms. Holmstorm et al. (28) observed no decrease in nasal resistance in 19% of their patients, while only 19% of patients reported no continuing symptoms. In the study by Piccino et al. (29), only 4% of patients had a decrease in airway resistance, whereas 88% reported a considerable subjective recovery. 

In contrast to the results of the present study, Kjaer Kard (30) reported a strong relationship between the personal sensation of nasal obstruction and the measurement of cross-sectional area, space, and airflow in a study in 2523 patients, although the correlation coefficient was low. However, a major limitation of this study was an error in the selection of the subjects. Many of the patients were male who had been referred to the doctor for sleeping disease or chronic respiratory complaints. Also, the study population included a high number of smokers.

## Conclusion

Although non-objective symptoms of nasal obstruction can improve considerably after surgery, no significant relationship was found with improvement in objective tests using rhinomanometry and acoustic rhinometry. Objective improvement in the openness of the nose is important because the nose serves as an airway, and rhinomanometry and acoustic rhinometry can therefore be helpful in the evaluation of septoplasty effectiveness. It is hard to diagnose nasal obstruction symptoms by measuring general nasal resistance and the cross-sectional area. Therefore, the variations in nasal obstruction symptoms are loosely reflected by variations in nasal resistance and the cross-sectional area. Although rhinomanometry is an appropriate method of determining the size of the obstruction by pathologic factors, clinical evaluation (rhinoscopy) and nasal endoscopy are currently the most common ways for the diagnosis and treatment of nasal obstruction, and objective tests do not offer a diagnostic value of surgery. Therefore, there is still a need for further investigation to explore other simple, cost-effective, clinically-oriented objective methods.
